# A call for benchmarking transposable element annotation methods

**DOI:** 10.1186/s13100-015-0044-6

**Published:** 2015-08-04

**Authors:** Douglas R. Hoen, Glenn Hickey, Guillaume Bourque, Josep Casacuberta, Richard Cordaux, Cédric Feschotte, Anna-Sophie Fiston-Lavier, Aurélie Hua-Van, Robert Hubley, Aurélie Kapusta, Emmanuelle Lerat, Florian Maumus, David D. Pollock, Hadi Quesneville, Arian Smit, Travis J. Wheeler, Thomas E. Bureau, Mathieu Blanchette

**Affiliations:** School of Computer Science, McGill University, McConnell Engineering Bldg., Rm. 318, 3480 Rue University, Montréal, Québec H3A 0E9 Canada; Department of Biology, McGill University, Stewart Biology Bldg., 1205 Ave. du Docteur-Penfield, Montréal, Québec H3A 1B1 Canada; McGill Centre for Bioinformatics, McGill University, Montréal, Québec Canada; Department of Human Genetics, McGill University, Montréal, Québec Canada; McGill University and Génome Québec Innovation Center, Montréal, Québec Canada; Centre for Research in Agricultural Genomics CSIC-IRTA-UAB-UB, 08193 Barcelona, Spain; Université de Poitiers, UMR CNRS 7267 Ecologie et Biologie des Interactions, Equipe Ecologie Evolution Symbiose, 5 Rue Albert Turpin, 86073 Poitiers Cedex 9, France; Department of Human Genetics, University of Utah School of Medicine, Salt Lake City, UT 84112 USA; Institut des Sciences de l’Evolution de Montpellier (ISE-M), Equipe Evolution, Vecteurs, Adaptation et Symbiose, UMR5554 CNRS-Université Montpellier, Montpellier, 34090 cedex 05 France; Laboratoire Evolution, Génomes, Comportement Ecologie, CNRS—Université Paris-Sud (UMR 9191)—IRD (UMR 247)—Université Paris-Saclay, F-91198 Gif-sur-Yvette, France; Institute for Systems Biology, 401 Terry Ave. N, Seattle, WA 98109 USA; Laboratoire Biometrie et Biologie Evolutive, Universite Claude Bernard—Lyon 1, UMR-CNRS 5558—Bat. Mendel, 43 bd du 11 novembre 1918, 69622 Villeurbanne cedex, France; INRA, UR1164 URGI—Research Unit in Genomics-Info, INRA de Versailles-Grignon, Route de Saint-Cyr, Versailles, 78026 France; University of Colorado School of Medicine, Aurora, CO 80045 USA; Department of Computer Science, University of Montana, Missoula, MT 59812 USA

## Abstract

DNA derived from transposable elements (TEs) constitutes large parts of the genomes of complex eukaryotes, with major impacts not only on genomic research but also on how organisms evolve and function. Although a variety of methods and tools have been developed to detect and annotate TEs, there are as yet no standard benchmarks—that is, no standard way to measure or compare their accuracy. This lack of accuracy assessment calls into question conclusions from a wide range of research that depends explicitly or implicitly on TE annotation. In the absence of standard benchmarks, toolmakers are impeded in improving their tools, annotators cannot properly assess which tools might best suit their needs, and downstream researchers cannot judge how accuracy limitations might impact their studies. We therefore propose that the TE research community create and adopt standard TE annotation benchmarks, and we call for other researchers to join the authors in making this long-overdue effort a success.

## Why does transposable element annotation matter, and why is it difficult?

Transposable elements (TEs) are segments of DNA that self-replicate in a genome. DNA segments that originated from TE duplications may or may not remain transpositionally active but are herein referred to simply as TEs. TEs form vast families of interspersed repeats and constitute large parts of eukaryotic genomes, for example, over half of the human genome [1–3] and over four fifths of the maize genome [4]. The repetitive nature of TEs confounds many types of studies, such as gene prediction, variant calling (i.e., the identification of sequence variants such as SNPs or indels), RNA-Seq analysis, and genome alignment. Yet their mobility and repetitiveness also endow TEs with the capacity to contribute to diverse aspects of biology, from disease [5], to genome evolution [6–8], organismal development [9], and gene regulation [10]. In addition to dramatically affecting genome size, structure (e.g., chromatin organization), variation (e.g., copy-number variation), and chromosome maintenance (e.g., centromere and telomere maintenance) [11], TEs also provide the raw material for evolutionary innovation, such as the formation of new protein-coding genes [12, 13], non-coding RNAs [14–16], and transcription factor binding sites [17, 18]. With the growing deluge of genomic data, it is becoming increasingly critical that researchers be able to accurately and automatically identify TEs in genomic sequences.

Accurately detecting and annotating TEs are difficult because of their great diversity, both within and among genomes. There are many types of TE [19, 20], which differ across multiple attributes, including transposition mechanism, TE structure, sequence, length, repetitiveness, and chromosomal distribution. Moreover, while recently inserted TEs have relatively low within-family variability, over time TE instances (specific copies) accumulate mutations and diverge, becoming ever more difficult to detect. Indeed, much of the DNA with as yet unknown origins in some genomes (e.g., human) might be highly decayed TE remnants [2, 8]. Because of this great diversity TEs within and among genomes, the primary obstacles to accurately annotating TEs vary dramatically among genomes, which have different TE silencing systems and which have undergone different patterns of TE activity and turnover. For instance, in some genomes (e.g., human [1]) the majority of TE-derived DNA is remnant of ancient bursts in the activity of just a few TE families; thus, annotation is mainly hampered by the high divergence of old and decayed TE copies, as well as extensive fragmentation of individual copies and the complex evolution of the TEs in the genome [6]. Other genomes (e.g., maize [4]) contain a large variety of recently active TEs; thus, defining and classifying the diverse families poses a considerable annotation challenge, as well as disentangling the complex and heterogeneous structures formed by clusters of TEs, such as internal deletions, nested insertions, and other rearrangements [21]. Furthermore, although libraries of known TE sequences are definitely useful, the TE families that are present in even closely related genomes may differ greatly [22], limiting the utility of such libraries in annotating newly sequenced genomes. Additional challenges to accurate annotation arise from multi-copy non-TE (host) gene families and segmental duplications, which in both cases mimic TEs because of their repetitiveness. Low complexity sequences and simple repeats may also be major sources of false positives [23]. Together, these issues pose considerable challenges to accurate, automated TE annotation.

Although the field of TE annotation may be broadly defined to include various activities, such as the identification and classification of TE families [19, 20], herein, we mainly discuss the detection and annotation of TE instances, particularly within assembled genomes, and the computational tools used to do so. A number of computational approaches and tools have been developed to identify TEs in assembled genomes. The two main approaches used currently are homology-based approaches, which use similarity to known TEs, and de novo approaches, which are typically based either on repetitiveness or on structural signatures (e.g., long terminal repeats or terminal inverted repeats) (reviewed in [24–26]). In addition, approaches are being developed to detect TEs using comparative genomics (e.g., insertion polymorphisms) [27] (Hickey et al., *pers. comm.*) or other properties such as the production of specific populations of small RNAs (e.g., siRNAs, piRNAs) [28]. However, to annotate assembled genomes, most researchers have implicitly adopted a de facto standard of tool use that incorporates just a fraction of available tools (Table 1), as follows: (i) Mask simple repeats (e.g., TRF [29]); (ii) Generate a library of ostensible TE sequences using repetitiveness-based tools (e.g., RepeatModeler, RepeatScout [30–32]), often augmented with one or more structure-based programs (e.g., LTR_FINDER [33], LTR_STRUC [34], or MITE-Hunter [35]); (iii) Classify consensus sequences into families (e.g., RepeatModeler [30] or RepClass [36]); (iv) Combine with an existing library of TE consensus sequences (or models) (e.g., RepBase [37] or recently Dfam [3]); (v) Finally, align the TE consensus sequences (or models) to the genome (e.g., either RepeatMasker [38] or Censor [39] with dependencies on sequence similarity tools such as cross_match [40], BLAST [41, 42], or nhmmer [43]). Different annotators often use and combine the tools in different ways, using different settings and ad hoc results filtering, library merging, and manual steps. A few groups have developed more complete pipelines that combine a wider selection of tools in a consistent manner (e.g., REPET [44]). A growing number of tools also operate directly on unassembled short genomic reads [45–50]. Finally, there are a small number of groups using largely manual methods to refine the libraries generated by these automated pipelines to create high quality TE libraries (Table 1) [3, 37, 51].

**Table 1:** Tools and databases used to annotate TEs in the genomes of multicellular eukaryotes published in 2014

Genome		Homology-based				De novo						Pipeline			
		Repbase	RepeatMasker	CENSOR	RepeatProteinMask	RepeatModeler	RepeatScout	PILER	LTR_FINDER	LTR_STRUC	MITE-Hunter	REPET	Other Databases	Other tools^a^	Ref.
*Phalaenopsis equestris* (tropical epiphytic orchid)	Plant (monocot)	✓	✓		✓		✓	✓	✓	✓					[81]
*Cyprinus carpio* (common carp)	Animal (bony fish)	✓	✓			✓								TEClass	[82]
*Esox lucius* (northern pike)	Animal (bony fish)	✓		✓							✓		Genbank, UniprotKB/SwissProt	Custom	[83]
*Oryza glaberrima* (African rice)	Plant (monocot)	✓	✓				✓		✓				MSU Repeats, custom (rice-specific)	Custom	[84]
*Callithrix jacchus* (common marmoset)	Animal (primate)	✓	✓												[85]
*Gossypium arboreum* (cultivated cotton)	Plant (dicot)	✓	✓		✓	✓	✓	✓	✓	✓					[86]
*Nicotiana tabacum* (common tobacco)	Plant (dicot)		✓				✓						TIGR, SGN (Solanaceae-specific)		[87]
*Glossina morsitans* (tsetse fly)	Animal (insect)		✓			✓	✓					✓	Genbank	RECON, TARGeT	[88]
*Oncorhynchus mykiss* (rainbow trout)	Animal (bony fish)	✓	✓	✓		✓			✓					E-inverted, Manual	[89]
*Tetrao tetrix* (black grouse)	Animal (bird)	✓	✓												[90]
*Pinus taeda* (loblolly pine)	Plant (gymnosperm)		✓	✓				✓			✓	✓	PIER 2.0 (conifer-specific)	Custom	[91]
*Spirodela polyrhiza* (duckweed)	Plant (monocot)		✓							✓			MipsREdat, MIPS PlantsDB	Custom	[92]
*Cynoglossus semilaevis* (half-smooth tongue sole)	Animal (flatfish)		✓				✓	✓	✓				RepBase (for classification)	E-inverted, Custom	[93]
*Capsicum annuum* L. and var. *glabriusculum* (cult. and wild peppers)	Plant (dicot)	✓	✓		✓	✓			✓				MSU repeats		[94]
*Capsicum annuum cv. CM334* (hot pepper)	Plant (dicot)	✓	✓			✓									[95]
*Anopheles sinensis* (mosquito)	Animal (insect)	✓	✓										Efam (mosquito-specific)		[96]

^a^Not all tools used in building TE libraries are listed (e.g., UCLUST, MUSCLE)

## Why do we urgently need benchmarks?

**Fig. 1:**
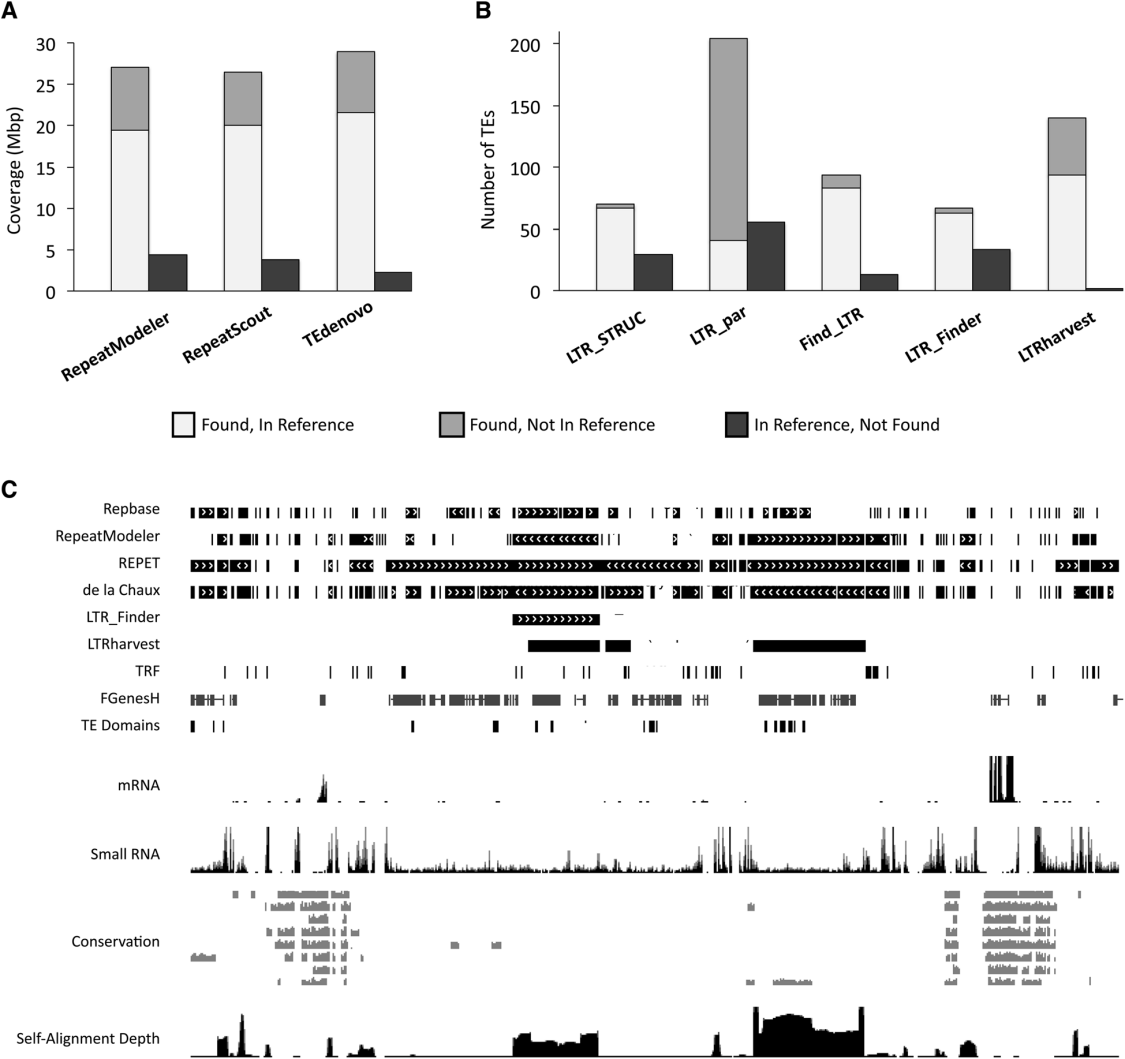
Variation among TE annotation tools. **a** TE coverage in the *Arabidopsis thaliana* genome resulting from three commonly used repetitiveness-based de novo tools, compared to a reference set of TEs [8]. The total amount of TE coverage differs between the three, as does the fraction of the reference TEs that were found or missed and the amount of non-reference putative TEs. **b** Full-length LTR TEs in the *Drosophila melanogaster* X chromosome found by five different LTR-specific de novo tools, compared to a reference set of TEs [24]. Similar to **a** but even more pronounced, the number of TEs found by the tools and their agreement with the reference set vary widely. **c** A 100-kbp segment of the *Arabidopsis lyrata* genome (scaffold_1:14,957,501-15,057,500) displayed on a custom UCSC genome browser [76, 77], illustrating differences among TE annotations resulting from several approaches, as well as additional genomic data useful in identifying bona fide TEs. From top to bottom, the tracks represent: RepeatMasker annotations using libraries from Repbase [37], RepeatModeler [30], REPET [44], or de la Chaux et al. [78]; full-length LTR TE predictions by LTR_Finder [33] or LTRharvest [79]; tandem repeat predictions by TRF [29]; gene models predictions by FGenesH [80]; a set of TE-specific domains [13]; mapped mRNA and small RNA short reads [77]; inter-species conservation (alignment percent identity plots) to other Brassicaceae species [77]; and genome self-alignment depth (generated with LASTZ)

TE predictions made by various methods are often quite divergent, with different tools having different strengths and weaknesses, competencies, and complementarities [8, 24, 52, 53] (Fig. 1). Why then are so few tools commonly used? How optimal are the various combinations of tools that are used? Most importantly, how accurate are the TE annotations that are produced?

In related disciplines including genome assembly [54], multiple sequence alignment [55–57], variant calling [58, 59], and cancer genomics [60], standard benchmarks have been successfully employed to measure and improve the accuracy of computational tools and methodologies. For example, in the area of protein structure prediction, researchers have taken great efforts to tackle the benchmarking problem for over 20 years [61].

However, for TE annotation, there is currently no standard way to measure or compare the accuracy of particular methods or algorithms. In general, there is a tradeoff between increased rates of true vs. false positives, both between different tools and between different settings for any given tool, a tradeoff that should ideally be optimized for each study. For instance, a study attempting to describe reasonable upper bounds of TE contributions to genome size might benefit from increased sensitivity (at the cost of specificity), while a study attempting to identify high stringency TE-derived regulatory regions might benefit from the converse. Regardless of the approach chosen for a study—even if it is a de facto standard tool with default settings—the resultant tradeoff between false and true positives ought to be quantified and reported. However, the current state of TE annotation does not facilitate such distinctions, especially for non-experts. Instead, it is left up to individual toolmakers, prospective tool users, or even downstream researchers to evaluate annotation accuracy. A few toolmakers with sufficient resources do invest the significant amount of effort required to assemble their own (often unpublished) test data sets and evaluate the accuracy of their tools. But for many toolmakers and most users, it is in practice too onerous to properly assess which methods, tools, and parameters may best suit their needs. The absence of standard benchmarks is thus an impediment to innovation because it reduces toolmakers’ ability and motivation to develop new and more accurate tools or to improve the accuracy of existing tools. Perhaps most importantly, the absence of benchmarks thwarts debate over TE annotation accuracy because there simply is little data to discuss. This lack of debate has the insidious effect that many of the ultimate end-users of TE annotation, researchers in the broader genomics, and genetics community who are not TE experts are left largely unaware of the complexities and pitfalls of TE annotation. These downstream researchers thus often simply ignore the impact of TE annotation quality on their results, leading to potentially avoidable problems, such as failed experiments or invalid conclusions. Thus, the lack of TE annotation benchmarks hinders the progress of not only TE research but also genomics and related fields in general.

At a recent conference at McGill University’s Bellairs Research Institute (St. James Parish, Barbados), a group of TE annotation and tools experts, including the authors, met to discuss these issues. We identified, as a cornerstone of future improvements to computational TE identification systems, a pressing need to create and to widely adopt benchmarks to measure the accuracy of TE annotation methods and tools and to facilitate meaningful comparisons between them. To clarify, we propose to generate benchmarks for genomic TE annotations, not intermediate steps such as library creation, although the latter would also be interesting to benchmark eventually. Benchmark creation will help alleviate all of the aforementioned issues. It will enable tool users to choose the best available tool(s) for their studies and to produce more accurate results, and it will democratize access, encouraging tool creation by additional researchers, particularly those with limited resources. Establishing benchmarks might also encourage the development of experimental pipelines to validate computational TE predictions. Perhaps most importantly, the adoption of standard benchmarks will increase transparency and accessibility, stimulating debate and leading the broader genomics-related research community towards an improved understanding of TEs and TE annotation. Thus, creating benchmarks may lead not only to improved annotation accuracy but may help to demystify a critical area of research that, relative to its importance, is often neglected and misinterpreted. We therefore believe that the TE research community should resolve to agree upon, create, and adopt standard sets of TE annotation benchmarks.

## What might TE annotation benchmarks consist of?

One of the reasons the TE annotation community still does not have accepted benchmarks may be that creating them is more challenging than in other fields. There are many possibilities for the form of such benchmarks and how they could be created. Ideally, they would consist of diverse, perfectly annotated, real genomic sequences; however, irrespective of the efforts made, a perfect TE annotation is impossible to achieve because it is irrevocably based on and limited by current TE detection methods. For instance, greatly decayed and rare TEs are difficult to detect and thus are sources of false negatives. Furthermore, highly heterogeneous TEs can be difficult to accurately assign to families, especially when they are decayed. To illustrate the potential extent of the first of these sources, it is likely that much of the unannotated part (about 40 %) of the human genome is comprised of ancient TE relics that are too diverged from each other to be currently recognized as such [1, 2, 8, 62, 63]. At a smaller scale, low copy-number TEs are missed by methods that rely on repetitiveness, including most tools used for building repeat libraries, but could be (originally) detected by structural signatures or by approaches using comparative genomics or other genomic attributes. An example of problematic TEs with ill-defined and highly heterogeneous structure is the helitron superfamily. Helitrons were initially discovered by computational analysis, based on the repetitiveness of some helitron families and the presence of genes and structural features not found in other TEs [64]. Although some families in some genomes can be detected through repetitiveness, in general, helitrons are especially difficult to detect because they do not have strong structural signatures, are often quite large, lack “canonical” TE genes, and conversely often do contain segments of low copy-number, non-TE (transduplicated) genome sequence [65–67]. Yet in many species, helitrons represent one of the most frequent types of TEs in the genome [64, 68–70]. In general, such false negatives in annotated real genomic data are a problem for benchmarking, since tools that manage to detect true TEs missing from the benchmark would be wrongly penalized. Conversely, false positives present in the benchmark would penalize tools with improved specificity. Ideally, the benchmarks would provide support for probabilistic annotations in order to help account for such uncertainties.

To overcome such issues with annotated genomic sequences, various approaches can be used. False negatives can be predicted by placing fragments of known TEs into real or synthetic genomes, an approach that is especially important for fragmented and degraded TEs [2]. False negatives caused by TE degradation can also be predicted using real genome sequences with known TEs that have been modified in silico by context sensitive evolutionary models [71]. False positive prediction is perhaps a more difficult problem. Because we do not have real genomic regions that we are certain have not been derived from TEs, a variety of methods have been used to produce false-positive benchmarks in which no true TE instances are expected to be found. These include reversing (but not complementing) real genomic sequence [3, 72] (which is also useful for detecting false extensions, i.e., predicted boundaries that extend beyond actual TEs [73]), shuffling real sequence while preserving mono- or di-nucleotide frequencies [2], and generating sequence using higher-order models [74]. Higher-order models may incorporate multiple key aspects of genome composition, complexity, and repeats, such as the diversity of TEs and their insertion patterns, the distribution of simple repeats and GC-content (compositional domains), varying rates of TE deletion, and other evolutionary processes [75]. Finally, it is important in any of these analyses to distinguish false positives (sequences that may have been generated by chance from mutation processes) from mis-annotation (sequences derived from other repetitive sequence or other TEs than the one being considered).

Even greater challenges are to predict mis-annotation or compound annotation of gene-like sequences that may be derived from TEs, as well as low complexity regions (e.g., CpG islands, pyrimidine stretches, and AT-rich regions) [74]. Another serious challenge is to avoid creating biases either for or against the methods used to initially identify any TEs incorporated into the models; for instance, if a certain tool originally identified a TE sequence, then that tool may have an advantage in accurately (re-) identifying the TE in a simulated genome. Furthermore, simulated genomes are not currently useful in evaluating TE annotation methods that employ additional types of data that are impractical to simulate, such as comparative genomic data or realistic populations of small RNA sequences. Finally and most fundamentally, the unknown cannot be modeled, and much about TE sequences, how they transpose, and how they evolve remains unknown. We need to consider, for example, how much our techniques are biased towards the types of TEs present in taxa that we have studied most intensively (e.g., mammals) and against TEs that have evolved in under-represented genomes. Thus, in designing and using standard benchmarks, we must remain cognizant that while improving our ability to detect and annotate TEs, they will also be ultimately limited by current knowledge of TEs and genome evolution.

Although this article is intended to promote discussion rather than providing ultimate solutions, we believe that an ideal benchmark data set would be as follows:

- Contributed, vetted, and periodically revised by the TE annotation community;
- Publicly available;
- A mixture of different types of simulated sequences and well-annotated real genomic regions;
- Sufficiently large in size to allow accurate assessment of tool performance;
- Representative of the biological diversity of genomes (e.g., size, TE density and family representation, evolutionary rates, and GC-content);
- Representative of the various states of assembly of ongoing genome sequencing projects;
- Accompanied by open-source support software that provides both online methods and an application programming interface (API) to compute a range of detailed meaningful statistics on the agreement between a user’s annotation and the benchmark data set;
- Eventually, provide support for probabilistic annotations that represent uncertainties, both at the level of the benchmark itself and user submitted annotations.

## Why and how should researchers contribute?

The success of this effort depends on buy-in from the TE community to create and contribute benchmark data sets, to use them in their own work, and to promote their adoption. Because of the multiple challenges involved in the creation of these benchmarks, it is unlikely that any first version will be completely satisfactory; however, this should not be used as an argument to dismiss this type of effort but rather to contribute to its improvement. In the coming months, we would like to initiate discussions with the wider TE community on the ideal format of a first set of TE benchmarks and to begin collecting data sets. We invite the entire TE research community to join us in this effort by providing feedback on the issues raised in this article, by commenting on specific benchmark data set proposals as they are made available, and by contributing their own benchmark data set proposals. To do so, please visit the project’s website at http://cgl.cs.mcgill.ca/transposable-element-benchmarking, or contact the authors.

## Abbreviations

API: application programming interface
LTR: long terminal repeat
TE: transposable element or DNA originating from them
